# A single-cycle replicable Rift Valley fever phlebovirus vaccine carrying a mutated NSs confers full protection from lethal challenge in mice

**DOI:** 10.1038/s41598-018-35472-7

**Published:** 2018-11-20

**Authors:** Kaori Terasaki, Terry L. Juelich, Jennifer K. Smith, Birte Kalveram, David D. Perez, Alexander N. Freiberg, Shinji Makino

**Affiliations:** 1 Department of Microbiology and Immunology, The University of Texas Medical Branch, Galveston, Texas 77555-1019 United States; 2 Department of Pathology, The University of Texas Medical Branch, Galveston, Texas 77555-1019 United States; 3 Center for Biodefense and Emerging Infectious Diseases, The University of Texas Medical Branch, Galveston, Texas 77555-1019 United States; 4 UTMB Center for Tropical Diseases, The University of Texas Medical Branch, Galveston, Texas 77555-1019 United States; 5 The Sealy Institute for Vaccine Sciences, The University of Texas Medical Branch, Galveston, Texas 77555-1019 United States; 6 Institute for Human Infection and Immunity, The University of Texas Medical Branch, Galveston, Texas 77555-1019 United States

## Abstract

Rift Valley fever phlebovirus (RVFV) is a pathogen of Rift Valley fever, which is a mosquito-borne zoonotic disease for domestic livestock and humans in African countries. Currently, no approved vaccine is available for use in non-endemic areas. The MP-12 strain is so far the best live attenuated RVFV vaccine candidate because of its good protective efficacy in animal models. However, there are safety concerns for use of MP-12 in humans. We previously developed a single-cycle replicable MP-12 (scMP-12) which lacks NSs gene and undergoes only a single round of viral replication because of its impaired ability to induce membrane-membrane fusion. In the present study, we generated an scMP-12 mutant (scMP-12-mutNSs) carrying a mutant NSs, which degrades double-stranded RNA-dependent protein kinase R but does not inhibit host transcription. Immunization of mice with a single dose (10^5^ PFU) of scMP-12-mutNSs elicited RVFV neutralizing antibodies and high titers of anti-N IgG production and fully protected the mice from lethal wild-type RVFV challenge. Immunogenicity and protective efficacy of scMP-12-mutNSs were better than scMP-12, demonstrating that scMP-12-mutNSs is a more efficacious vaccine candidate than scMP-12. Furthermore, our data suggested that RVFV vaccine efficacy can be improved by using this specific NSs mutant.

## Introduction

RVFV is an arbovirus of major public health concern in African and Middle Eastern countries. The virus belongs to family *Phenuiviridae*, genus *Phlebovirus*, and has a genome composed of three single-stranded, negative-sense RNA segments; L, M, and S^1^. The L segment encodes a viral RNA-dependent RNA polymerase (L protein). The M segment encodes two accessory proteins, 78-kDa and NSm proteins, and two major viral envelope proteins, Gn and Gc, the latter of which carries a fusion peptide and induces membrane fusion^2,3^. The S segment uses an ambi-sense strategy to express the nucleo capsid (N) protein and an accessory protein, NSs. NSs is a major viral virulence factor and has multiple biological functions that are important for countering the host antiviral responses. NSs suppresses general transcription^4–6^ and IFN-β mRNA transcription^7^, and promotes the degradation of double-stranded RNA-dependent protein kinase R (PKR), an antiviral IFN-stimulated gene product, to prevent phosphorylation of eIF2α triggered by RVFV infection^8–11^.

RVFV circulates among ruminants and mosquitoes and has been repeatedly causing outbreaks in countries where the disease is endemic. Heavy rainfall and flooding are considered to be associated with the outbreaks. As RVFV infects various ubiquitous species of mosquitoes^12,13^, there is an increasing concern that the virus can invade other regions of the world by the enhanced spread of mosquitoes due to climate changes^14,15^. In fact, RVFV has already seeped beyond Africa^16,17^. There is also the potential for RVFV to be used as a bioterrorism agent, which could result in its spread to other countries.

Human RVFV infections generally manifest as self-limiting and nonfatal illnesses. However, a small percentage of patients develop encephalitis, permanent vision loss, and hemorrhagic fever with a high mortality rate and also suffer from long-term neurological symptoms^18,19^. In domestic ruminants, RVFV infection causes high mortality and spontaneous abortions with severe hepatic disease^20^. Age-dependent susceptibility to RVFV has been reported in rats and gerbils^21–23^. Consistent with this notion, RVFV infection causes high mortality rates in young ruminants^19,24^. Currently, there is no commercially available RVFV vaccine for human use in non-endemic countries.

Previous studies have shown that vaccination is an effective way to control the diseases caused by RVFV in animal models and also suggest that neutralizing antibodies play a major role in protection against RVFV (reviewed in^25,26^). Therefore, RVFV vaccine developments primarily focus on the efficient expression or delivery of Gn/Gc, which carry virus neutralizing epitopes^27^, in immunized animals to induce high titers of neutralizing antibodies. Additionally, the importance of anti-N protein antibody in RVFV vaccine efficacy has been shown; immunization of animals with purified N protein or DNA constructs and other viral platforms that encode RVFV N protein conferred partial protection against lethal RVFV challenge^28–34^. Virus-like particles (VLP) which carry L RNA and S-like RNA expressing N protein in infected cells showed better immunogenicity than irradiation-inactivated VLP, suggesting that the replication of viral RNA and/or N and L protein expression in infected cells enhanced immunogenicity^35^. Recent works^36,37^ also identified epitopes in N protein for CD4+ T cells, which play a role in the clearance of RVFV from infected tissues^38^, and for CD8+ T cells, which possibly activate cell mediated immunity upon RVFV infection.

MP-12 strain is a RVFV vaccine candidate, which was obtained by the serial passage of wild-type (wt) RVFV strain ZH548 in the presence of a chemical mutagen, 5-fluorouracil^39^. MP-12 is so far the most promising vaccine candidate (reviewed in^40^). However, there is a safety concern with the use of MP-12 for the immunization of the general public because of its residual virulence in animals. Intraperitoneal (i.p.) inoculation of young mice and SCID mice with MP-12 results in lethal outcomes^41,42^. MP-12 also causes disease in livestock, especially in young animals^43,44^. The effect of MP-12 vaccination in humans with an immature or impaired immune system, including children, remains largely unknown.

In an effort to balance the safety and protective efficacy of RVFV vaccine, we have developed a MP-12 variant, whose replication is limited to a single round in naïve cells. We named this MP-12 variant, single-cycle replicable MP-12 (scMP-12). The scMP-12 encodes a mutant Gc with impaired ability to induce membrane fusion and green fluorescent protein (GFP) in place of NSs protein^45^. The scMP-12 underwent multiple rounds of replication in Vero-derived cells, which stably express intact Gn/Gc (Vero-G cells), while it underwent single-cycle replication in naïve cells. Because of its single-cycle replication property, scMP-12 completely lacked neurovirulence in suckling mice. Vaccination of mice with 10^5^ PFU of the scMP-12 induced RVFV neutralizing antibodies and protected about 90% of them from a lethal RVFV challenge. Although scMP-12 was superior to MP-12 in safety, its immunogenicity and protective efficacies were not as high as those of MP-12 in mice. Accordingly, development of scMP-12 variants that elicit higher immunogenicity and/or protective efficacy is important.

The present study explored whether replacing GFP of scMP-12 with a specific NSs mutant improves immunogenicity and/or protective efficacy. Our past studies on characterization of naturally occurring NSs mutants showed that NSs carrying the R16H/M250K mutation (NSs R16H/M250K) lacked host transcription suppression function, moderately suppressed IFN-β transcription, and retained PKR degradation function^42^. In the present study, we generated a scMP-12 variant (scMP-12-mutNSs) carrying the NSs R16H/M250K and examined its replication properties in cell cultures, and its immunogenicity and protective efficacy in mice. Our study showed that scMP-12-mutNSs had better vaccine efficacy than scMP-12.

## Results

### Replication properties of scMP-12 and scMP-12-mutNSs in cell cultures

**Figure 1:**
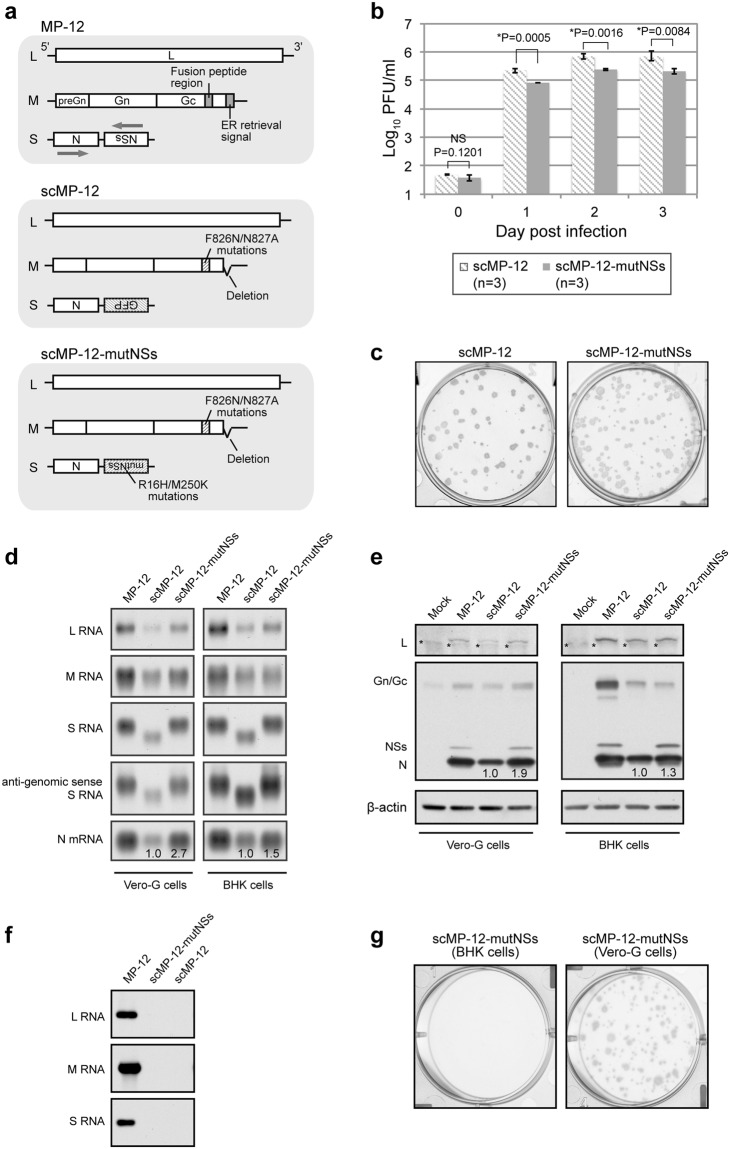
Replication properties of scMP-12 and scMP-12-mutNSs in cell cultures. (**a**) Schematic diagrams of the anti-genomic sense genomic RNAs of MP-12, scMP-12, and scMP-12-mutNSs. (**b**) Growth kinetics of the scMP-12 and scMP-12-mutNSs in Vero-G cells. Vero-G cells were infected with the indicated viruses at a moi of 0.05. Culture supernatant was collected at the indicated time points and infectivity determined by plaque assay, where viral plaques were stained by anti-N protein antibody, in Vero-G cells. The difference in virus titers between scMP-12 and scMP-12-mutNSs was assessed by unpaired t-test (two-tailed) at each time point. The data represents mean value ± standard deviation from three independent experiments. NS: no significant difference. *Significant difference. (**c**) Plaque morphologies of the scMP-12 and scMP-12-mutNSs in Vero-G cells. (**d**) Accumulation of viral RNAs in infected cells. Vero-G cells or BHK cells were infected with MP-12, scMP-12, or scMP-12-mutNSs at a moi of 0.1. Total intracellular RNA was extracted at 12 h p.i and analyzed by Northern blot. The numbers shown in the bottom panels indicate the relative band intensities of the N mRNA in scMP-12 and scMP-12-mutNSs-infected cells. The band intensity in scMP-12-infected cells was defined as 1.0. (**e**) Accumulation of viral proteins in infected cells. Vero-G cells or BHK cells were mock-infected (Mock) or infected with MP-12, scMP-12, or scMP-12-mutNSs at a moi of 0.1. Whole cell lysate collected at 12 h p.i was subjected to Western blot analysis using anti-L protein antibody^60^ (top panels), anti-MP-12 antibody (middle panels), and anti-β-actin (bottom panels). Asterisk indicates non-specific bands detected by anti-L antibody. The numbers in the middle panels indicate the relative band intensities of the N protein signals in scMP-12 and scMP-12-mutNSs-infected cells. The band intensity in scMP-12-infected cells was defined as 1.0. The data in (**d**) and (**e**) are representative data from three independent experiments. (**f**) Single-cycle property of scMP-12-mutNSs. Culture supernatant of BHK cells infected with MP-12, scMP-12, or scMP-12-mutNSs at a moi of 1 was collected at day 2 post-inoculation and inoculated into fresh BHK cells without dilution. Total RNA was extracted from the inoculated cells, which was harvested at 16 h p.i. The same amount of RNA were subjected to Northern blot analysis using RNA probes, which bind to viral genomic-sense L, M, or S RNA. (**d**) Single-cycle property of scMP-12-mutNSs. Left panel: Culture supernatant of scMP-12-mutNSs-infected BHK cells, which was used for (**f**), was inoculated into VeroE6 cells without dilution for plaque assay. Cells were stained by anti-N antibody at 3 days p.i. Right panel: scMP-12-mutNSs, which had undergone amplification in Vero-G cells, was inoculated into Vero-G cells and the plaques are stained by anti-N antibody at day 3 p.i. as a positive control.

The previously developed scMP-12 carried L RNA, S RNA that encoded N and GFP, and an M RNA mutant that carried the mutations F826N and N827A in the fusion peptide region^2,3^ of Gc^45,46^. It also had a deletion of the C-terminal 5 amino acids in Gc, which serves as an ER retrieval signal^47^ (Fig. 1a). The scMP-12 was safe, because of its single-cycle replicable property, and was also effective as a vaccine in the mouse model^45^. To improve the immunogenicity of scMP-12, we generated scMP-12-mutNSs, which differed from scMP-12 by encoding NSsR16H/M250K in place of GFP of scMP-12 (Fig. 1a), by using a reverse genetics system^48^. The scMP-12-mutNSs retained the introduced mutations in the NSs and Gc genes after five serial passages in Vero-G cells. Both scMP-12-mutNSs and scMP-12 showed similar growth kinetics in Vero-G cells, yet the titers of the former were, for unknown reasons, 2.6–3.6 fold lower than those of the latter at 1, 2, and 3 days p.i. (Fig. 1b). Both scMP-12 and scMP-12-mutNSs formed similar sized plaques in Vero-G cells (Fig. 1c), implying that scMP-12 and scMP-12-mutNs disseminated to a similar degree in this cell type. Northern blot analysis showed accumulation of higher amounts of N mRNA, which encodes N protein, in scMP-12-mutNSs-infected cells than in scMP-12-infected cells (Fig. 1d). Consistent with the higher accumulation level of N mRNA in scMP-12-mutNSs-infected cells, the amount of N protein was also higher in scMP-12-mutNSs-infected cells than in scMP-12-infected cells (Fig. 1e). We also observed a higher level of accumulation of S RNA and marginally higher levels of accumulation of other viral RNAs in scMP-12-mutNSs-infected cells than in scMP-12-infected cells (Fig. 1d). Because N protein is essential for viral RNA synthesis^49–51^, the higher level of N protein accumulation in scMP-12-mutNSs-infected cells likely promoted viral RNA synthesis. However, accumulation of L and Gn/Gc proteins were similar between scMP-12-mutNSs-infected cells and scMP-12-infected cells (Fig. 1e). To test the single round replication property of scMP-12-mutNSs, we inoculated culture supernatant of BHK cells, which had been infected with scMP-12-mutNSs, into fresh BHK cells and VeroE6 cells. Synthesis of viral RNAs did not occur in the infected BHK cells (Fig. 1f) and plaques were not detected by N protein staining in infected VeroE6 cells (Fig. 1g). These results confirmed the single-cycle replication property of scMP-12-mutNS.

### Effects of scMP-12-mutNSs replication on PKR levels and host transcription

**Figure 2:**
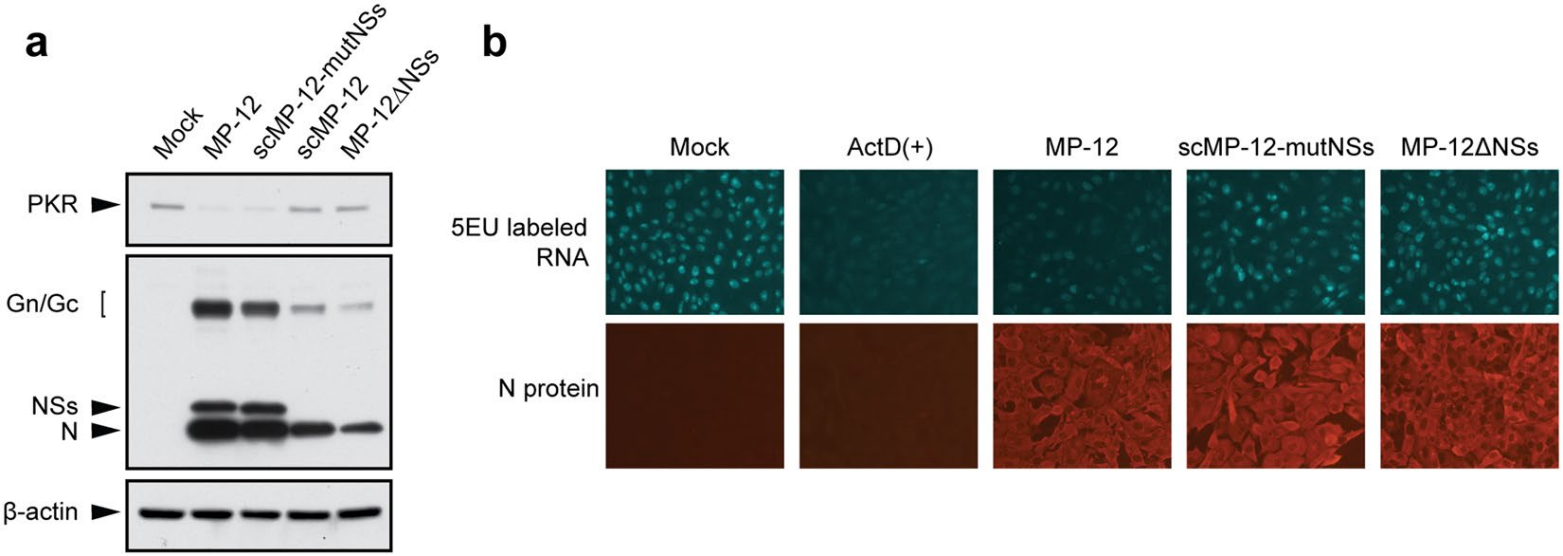
Effects of scMP-12-mutNSs replication on PKR abundance and host transcription. (**a**) Levels of total PKR in scMP-12-mutNSs-infected cells. HeLa cells were infected with indicated viruses at a m.o.i. of 1 and harvested at 8 h p.i. Cell lysates were subjected to Western blot analysis using anti-PKR antibody (top panel), anti-MP-12 antibody (middle panel), and anti-beta-actin antibody (bottom panel). (**b**) General RNA transcription in scMP-12-mutNSs-infected cells. Vero E6 cells were treated with 5EU for 1 h at 16 h after infection with indicated viruses at a moi of 1 and fixed with 4% paraformaldehyde. The cells were treated with Alexa Fluor 488 conjugated azide to visualize 5EU-labeled RNA and further stained by anti-N antibody followed by Alexa Fluor 594 conjugated secondary antibody (N protein).

MP-12 carrying the NSs mutant (R16H/M250K) is deficient for host general transcription suppression, yet it degrades PKR^42^. We investigated whether the mutant NSs also showed these biological functions in scMP-12-mutNSs-infected cells. Experiments using HeLa cells showed that the amounts of PKR in scMP-12-mutNSs-infected cells and MP-12-infected cells were similar and lower than those in mock-infected cells, suggesting that scMP-12-mutNSs induced PKR degradation (Fig. 2a). In contrast, reduction in the amounts of PKR did not occur in scMP-12-infected cells and cells infected with MP-12 lacking NSs gene (MP-12ΔNSs). We also noted less efficient accumulation of viral proteins of scMP-12 and MP-12ΔNSs, implying that these viruses lacking the NSs gene underwent less efficient virus replication due to induction of type I IFN production.

We analyzed host global transcription in scMP-12-mutNSs-infected cells by labeling newly synthesized RNA with 5-ethynyl uridine (5EU) at 16–17 h post-infection (Fig. 2b). As expected, mock-infected cells, but not actinomycin D-treated cells, showed strong fluorescent signals, which represented newly synthesized RNAs. MP-12ΔNSs-infected cells showed strong fluorescent signals, whereas low levels of fluorescent signals were detected in MP-12-infected cells, revealing the NSs-mediated host transcriptional suppression. The scMP-12-mutNSs-infected cells showed brighter fluorescent signals than MP-12-infected cells, suggesting that the mutant NSs did not inhibit host transcription. Taken together, these results suggested that like MP-12 carrying NSs with R16H/M250K mutation^42^, scMP-12-mutNSs failed to induce host transcriptional suppression, yet it induced PKR degradation in infected cells.

### Immunogenicity of scMP-12 and scMP-12-mutNSs

**Figure 3:**
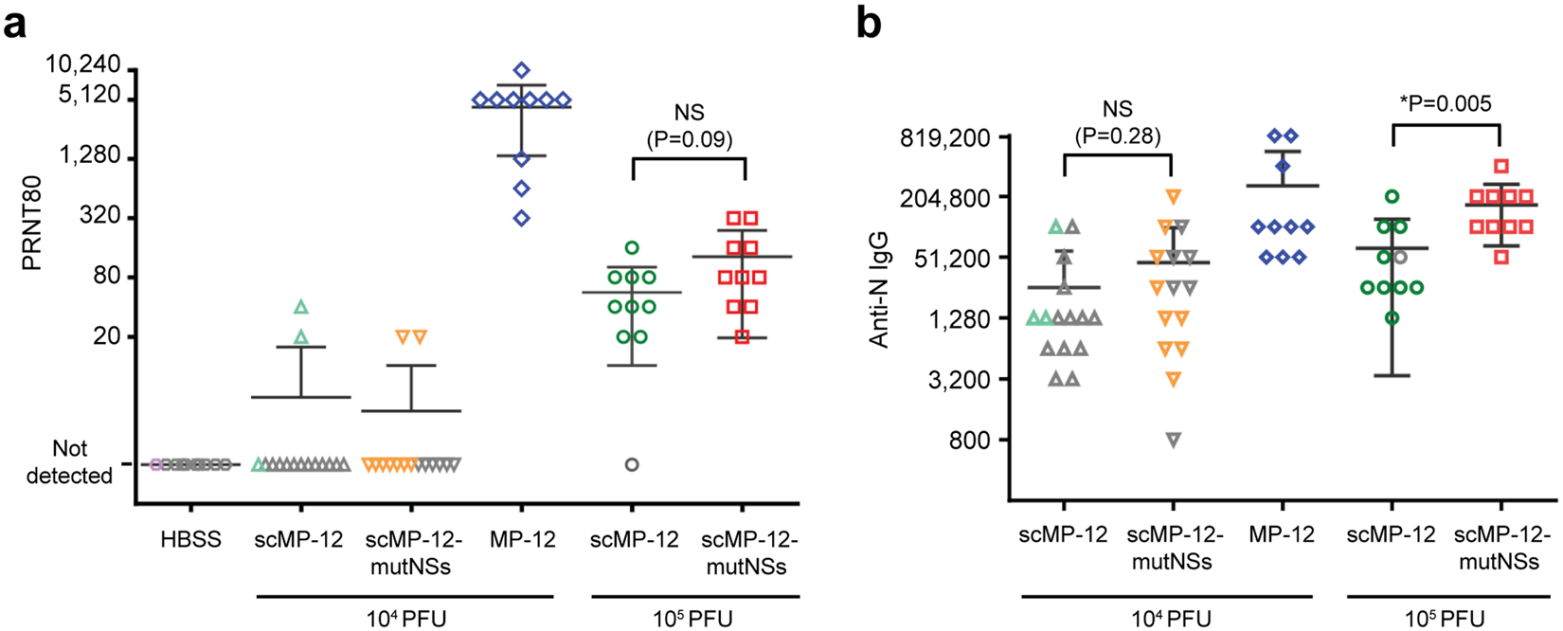
Immunogenicity of scMP-12 and scMP-12-mutNSs. Five-week-old CD-1 female mice were immunized with 10^4^ PFU of scMP-12, 10^4^ PFU of scMP-12-mutNSs, 10^4^ PFU of MP-12, 10^5^ PFU of scMP-12, 10^5^ PFU of scMP-12-mutNSs, or HBSS. All viruses were prepared in Vero-G cells. At 35 days post-immunization, sera were collected and subjected to PRNT80 and ELISA to detect RVFV neutralizing antibody and anti-N IgG, respectively. (**a**) Data in Fig. 2a is mean PRNT80 titer ± standard deviation (SD). Differences between 10^5^ PFU of scMP-12 and scMP-12-mutNSs were analyzed by Mann-Whitney U-test. (**b**) Mean anti-N antibody titer ± SD measured by ELISA. Differences between scMP-12 and scMP-12-mutNSs at the same dose were analyzed by Mann-Whitney U-test. NS: no significant difference. *Significant difference. Euthanized and dead animals after wt RVFV challenge are shown as gray colored symbols.

We compared the immunogenicity of scMP-12-mutNSs with scMP-12 in 5-week-old female CD1 mice intramuscularly injected with 10^4^ or 10^5^ PFU of either viral strain; inoculation with 10^4^ PFU of MP-12 or HBSS served as controls. Serum was collected from the immunized mice at 36 days p.i. The 80% plaque-reduction neutralization (PRNT80) titers and anti-N IgG titers of the mice sera at 36 days p.i. were shown in Fig. 3a and 3b, respectively, where each mark represents an individual animal. Grey marks represent mice which succumbed to wt RVFV challenge described below, while marks with other colors represent survivors.

No neutralizing antibody was detected in the HBSS-inoculated mice, and the mean titers of PRNT80 of those mice given 10^4^ PFU of MP-12 was 1:4,320. Mice immunized with 10^5^ PFU of scMP-12 and those immunized with 10^5^ PFU of scMP-12-mutNSs had PRNT80 titers of 1:56 and 1:130, respectively, although these titers were not statistically different (Fig. 3a). The majority of mice immunized with 10^4^ PFU of scMP-12 or scMP-12-mutNSs did not elicit detectable levels of neutralizing antibodies, except for two mice in each group, which were survivors in wt RVFV challenge experiments described below.

The mean titers of anti-N antibody were: 1:25,600 in scMP-12 10^4^ PFU group; 1:45,493 in scMP-12-mutNSs 10^4^ PFU group; 1:627,720 in scMP-12 10^5^ PFU group; 1:168,960 in scMP-12-mutNSs 10^5^ PFU group; and 1:261,120 in MP-12 10^4^ PFU group. The mice immunized with scMP-12-mutNSs showed overall higher anti-N antibody titers than those immunized with scMP-12 and the difference was significant between the 10^5^ PFU groups (Fig. 3b). Mice immunized with 10^5^ PFU of scMP-12-mutNSs induced comparable levels of anti-N antibody titers to those immunized with 10^4^ PFU of MP-12.

### Protective efficacy of scMP-12 and scMP-12-mutNSs

**Figure 4:**
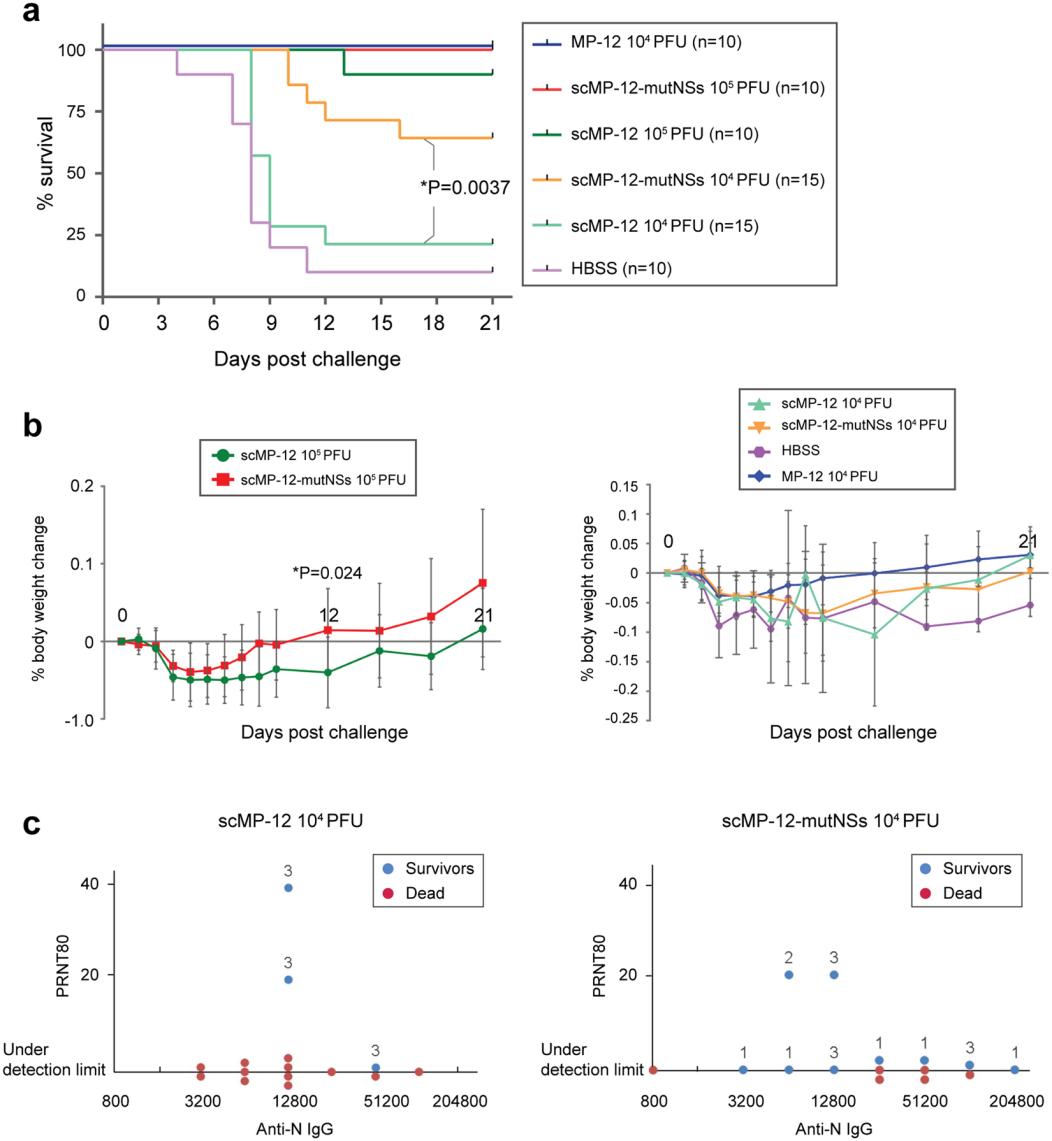
Protective efficacy of scMP-12 and scMP-12-mutNSs. Immunized mice shown in Fig. 3 were i.p. challenged with 10^3^ PFU of ZH501 at 40 days post-immunization and their body weight, clinical symptoms, and survival was monitored for 21 days. (**a**) Survival curves of mice immunized with the indicated virus after lethal RVFV challenge. Difference in the curves between 10^4^ PFU scMP-12 and 10^4^ PFU scMP-12-mutNSs groups were assessed by log-rank test. (**b**) Body weight changes of mice immunized with indicated viruses as a percentage compared to the day of challenge. Groups of 10^5^ PFU dose were shown in left panel. Groups of 10^4^ PFU dose and HBSS were shown in right panel. The data are mean ± SD. Differences of the body weight between 10^5^ PFU scMP-12 and 10^5^ PFU scMP-12-mutNSs were analyzed by unpaired t-test (two-tailed) at each time point. (**c**) PRNT80 titers, anti-N IgG titers, and survival of each individual mouse in 10^4^ PFU scMP-12-immunized (left panel) and 10^4^ PFU scMP-12-mutNSs-immunized (right panel) groups were plotted. Blue and red dots indicate survived and dead mice, respectively. The numbers above the dots represent the highest clinical scores of the survived mice during the challenge.

The immunized mice were challenged at 40 days post-immunization with 10^3^ PFU of the ZH501 RVFV strain via i.p. route, and were monitored for changes in survival (Fig. 4a) and body weight (Fig. 4b). For mice immunized with 10^4^ PFU of scMP-12-mutNSs or 10^4^ PFU of scMP-12, the relationship among PRNT80 titers, anti-N IgG titers, highest clinical scores, and survival or death of each mouse are shown in Fig. 4c.

Most of the HBSS-inoculated mice succumbed to wt RVFV by 11 days p.i., except for one, which showed clinical signs of disease with a clinical score of 3 and then recovered (Fig. 4a). All mice immunized with 10^4^ PFU of MP-12 survived without the signs of disease. Nine out of the 10 mice immunized with 10^5^ PFU of scMP-12 and all the mice immunized with 10^5^ PFU of scMP-12-mutNSs survived. All mice that survived in the 10^5^ PFU groups did not show any sign of disease throughout the study. Three out of the 15 mice immunized with 10^4^ PFU of scMP-12 survived, and all animals that survived showed signs of disease with clinical scores of 3 (Fig. 4a and c). In contrast, nine out of the 15 mice immunized with 10^4^ PFU of scMP-12-mutNSs survived. Among the survivors, five showed no sign of disease, whereas four had a clinical score ranging from 2 to 3 (Fig. 4a and c). Also, onset of death occurred earlier in scMP-12-immunized mice than in scMP-12-mutNSs-immunized mice. Survival curves of the 10^4^ PFU groups differed significantly (Fig. 4a). Overall, these data showed that the protective efficacy of scMP-12-mutNSs was higher than scMP-12.

We also observed a trend that mice immunized with 10^5^ PFU of scMP-12-mutNSs showed less pronounced weight loss than those immunized with 10^5^ PFU of scMP-12. The average body weight change, which was relative to body weight at day 0, at day 12 post-challenge was 1.5% in the scMP-12-mutNSs group and −4% in scMP-12 group. This difference was statistically significant (Fig. 4b left panel). The data suggested that 10^5^ PFU of scMP-12-mutNSs protected mice from weight loss induced by wt RVFV infection more efficiently than 10^5^ PFU of scMP-12. We did not see a clear trend in weight loss between mice immunized with 10^4^ PFU of scMP-12 and those immunized with 10^4^ PFU of scMP-12-mutNSs (Fig. 4b).

## Discussion

The present study examined the immunogenicity and protective efficacy of scMP-12-mutNSs using a mouse model. Immunization of mice with 10^5^ PFU of scMP-12-mutNSs induced production of RVFV neutralizing antibody and anti-N antibody, and conferred full protection of the immunized mice from lethal RVFV challenge. Mice immunized with 10^5^ PFU of scMP-12-mutNSs elicited higher anti-N antibody titers than those immunized with 10^5^ PFU of scMP-12. Furthermore, the number of mice that survived from wt RVFV challenge was significantly higher among mice immunized with 10^4^ PFU of scMP-12-mutNSs than those immunized with the same dose of scMP-12. Taken together, these data showed that scMP-12-mutNSs elicited better immunogenicity and demonstrated higher protective efficacy than scMP-12 in the mouse model.

We evaluated the immunogenicity of scMP-12 and scMP-12-mutNSs by measuring anti-N antibody and PRNT80 titers in the immunized mice. Mice immunized with 10^5^ PFU of scMP-12-mutNSs elicited statistically higher anti-N antibody titers than those immunized with the same dose of scMP-12 (Fig. 3b). Mice immunized with 10^4^ PFU scMP-12-mutNSs also had higher mean anti-N protein antibody titers than those immunized with 10^4^ PFU scMP-12, yet the difference was not statistically significant (Fig. 3b). Because the level of N protein accumulation was higher in scMP-12-mutNSs-infected cells than in scMP-12-infected cells (Fig. 1e), we speculate that it contributed to the efficient production of anti-N antibody in scMP-12-mutNSs-immunized mice. As N protein contains epitopes for CD4+ T cells^37^, the efficient N protein accumulation might have induced efficient activation of CD4+ T cells. This was likely followed by activation of B cells, leading to production of higher titers of anti-N antibodies in scMP-12-mutNSs-immunized mice than in scMP-12-immunized mice. In contrast to anti-N antibody titers, PRNT80 titers in mice immunized with 10^5^ PFU of scMP-12-mutNSs and in those immunized with the same titers of scMP-12 did not show statistical differences, although the former had higher mean PRNT80 titers than the latter. These data suggest that replacing the GFP gene of scMP-12 with NSsR16H/M250K had minor effects on the induction of PRNT80 titers in the mouse model.

Our data suggested a major contribution of the humoral immune response to the protection of the immunized mice following wt RVFV challenge. All mice with PRNT80 titers above 1:20 survived after wt RVFV challenge (Fig. 3a), and, in the 10^5^ PFU scMP-12 group, the only animal having an undetectable level of PRNT80 titer had a fatal outcome (Fig. 3a). These results support the notion that neutralizing antibodies play an important role in protection of animals from the disease. However, PRNT80 titers alone may not determine the protective efficacy, as one mouse immunized with 10^4^ PFU of scMP-12 and seven mice immunized with 10^4^ PFU of scMP-12-mutNSs survived from wt RVFV challenge without showing detectable levels of neutralizing antibodies. Furthermore, in the group of animals immunized with 10^4^ PFU of scMP-12-mutNSs, five of the seven animals survived and lacked detectable levels of neutralizing antibodies, but did not show any clinical signs following wt RVFV challenge (Fig. 4c). As previous studies demonstrated that immunization of mice with N protein conferred partial protection against virulent RVFV challenge^28–34^, anti-N antibodies potentially contributed to survival in these mice. However, there was no clear correlation between anti-N antibody titers and clinical outcomes, including change in body weight, clinical scores, and survival, in mice immunized with 10^4^ PFU of scMP-12 or scMP-12-mutNSs (Fig. 4c). Hence, it is conceivable that a combined effect of anti-N antibodies and low level of neutralizing antibodies (below detection limits in PRNT80 assays) conferred protection against disease. Another possibility is that the mice with low levels of total anti-N antibodies survived from wt RVFV due to production of sufficient amounts of highly protective anti-N antibodies, which recognize specific epitopes in the N protein.

Consistent with our previous study^42^, replication of scMP-12-mutNSs did not inhibit general host transcription, yet it induced PKR degradation (Fig. 2). As scMP-12-mutNSs showed higher protective efficacy than scMP-12, we speculate that the PKR degradation in scMP-12-mutNSs-infected cells contributed to the high protective efficacy. The NSs-induced PKR degradation prevents eIF2α phosphorylation and secures viral protein expression in RVFV-infected cells^8–11^, where NSs also suppresses host general transcription. The higher levels of N protein accumulation detected in scMP-12-mutNSs-infected cells than in scMP-12-infected cells (Fig. 1e) suggested that the NSs-mediated PKR degradation prevented eIF2α phosphorylation, leading to efficient N protein expression in scMP-12-mutNSs-infected cells. Conversely, in the absence of NSs expression, the replication of scMP-12 probably induced eIF2α phosphorylation in infected cells, resulting in less efficient accumulation of N protein. In support of this, Lihoradova *et al*. reported that MP-12 carrying a dominant negative PKR in place of NSs expressed higher levels of N protein than MP-12 lacking NSs in infected cells; both viruses did not inhibit host transcription and only the former virus inhibited PKR function^52^. Furthermore, the PKR degradation function of NSs possibly affected host protein expression by preventing eIF2α phosphorylation and maintaining a cellular environment suitable for host gene expression. It is conceivable that some host proteins, including proinflammatory cytokines and chemokines, whose expression was not suppressed in scMP-12-mutNSs-infected cells, facilitated induction of protective immune responses. Because PKR degradation does not occur in scMP-12-infected cells, expression of these putative host proteins in scMP-12-infected cells might not be as high as in scMP-12-mutNSs-infected cells. Taken together, our data and a previous study^52^ suggest that the PKR degradation function of the NSs enhances vaccine efficacy of RVFV. In contrast to the PKR degradation function, the NSs-mediated global transcription suppression would prevent synthesis of mRNAs encoding these putative host proteins that promote protective efficacy and would inhibit efficient accumulations of these proteins, possibly leading to attenuation of protective efficacy. In this regard, testing the protective efficacy of MP-12 mutant carrying NSsR16H/M250K would be meaningful, as the biological properties of NSsR16H/M250K may be superior to wt NSs for induction of protective immune responses in replication-competent virus as well.

As both scMP-12 and scMP-12-NSsmut were amplified in Vero-G cells and used for immunization, we also used MP-12 that underwent amplification in Vero-G cells for immunization in the present study. Unexpectedly, mice immunized with 10^4^ PFU of MP-12 induced a mean PRNT80 titer of 1:4,320 (Fig. 3a), which was significantly higher than those of 1:310 in mice immunized with the same dose of MP-12, which was prepared in VeroE6 cells, in our previous study^45^. Since expression of Gn and Gc proteins induces production of VLP^53,54^, there is a possibility that low levels of VLPs were released in Vero-G cells. If this is the case, MP-12 prepared in Vero-G cells had better immunogenicity than MP-12 prepared in VeroE6 cells due to the presence of VLPs, which also served as immunogens.

In summary, we have generated a single-cycle replicable RVFV MP-12, carrying a mutated NSs that lacks the ability to inhibit host transcription, as a safe and effective RVFV vaccine candidate. Further modification that increases the infectivity of our new scMP-12 would be valuable for the practical use of single-cycle replicable MP-12 and its variants in humans.

## Materials and Methods

### Ethics statement

All experiments using mice in this study were conducted in facilities certified by the Association for Assessment and Accreditation of Laboratory Animal Care in accordance with the Animal Welfare Act, NIH guidelines, and U.S. federal law. The animal protocol was approved by the UTMB Institutional Animal Care and Use Committee. All work with the wild type RVFV ZH501 strain was conducted in the Robert E. Shope BSL-4 laboratory at the University of Texas Medical Branch (UTMB) in accordance with NIH guidelines and U.S. federal law.

### Cells and viruses

Maintenance of the cell line which stably expresses T7 RNA polymerase, BSR-T7/5 cells^55^, was previously described^56^. Vero-G cells^45^, which stably express RVFV Gn and Gc proteins, were maintained in Dulbecco’s Modified Eagle Medium supplemented with 10% fetal bovine serum (FBS), 10 μg/ml of blasticidine (Invitrogen), and antibiotics. VeroE6 cells were maintained in Dulbecco’s minimal essential medium (MEM) with 5% FBS and antibiotics. BHK-21 cells were grown in MEM alpha medium (Gibco) with 5% FBS and antibiotics. The MP-12 strain of RVFV was rescued by reverse genetics as previously described^48^ and propagated in Vero-G cells. RVFV ZH501 strain^57^ was prepared in VP-SFM (Thermo fisher scientific) supplemented with 2 mM L-glutamine.

### Rescue and amplification of scMP-12 and scMP-12-mutNSs

scMP-12 and scMP-12-mutNSs were generated as described previously^42,45^. Briefly, for recovery of scMP-12, BSR-T7/5 cells were co-transfected with T7-driven plasmids encoding L and N proteins^48^ and pCAGGS-G encoding bovine codon optimized sequence for Gn/Gc protein expression^45^, and T7-driven viral RNA expression plasmids^48^ encoding the L RNA, scMP-12 M RNA, which carries two amino-acid substitutions, F826N and N827A, and 5 amino acid deletion of C-terminal end of Gc^45^, and S RNA encoding N and GFP. scMP-12-mutNSs was rescued using essentially the same method, except for using S RNA encoding N and NSsR16H/M250K. Culture fluids from the transfected cells were collected at 5 to 7 days post-transfection (P0 samples) and inoculated into Vero-G cells for amplification of scMP-12 or scMP-12-mutNSs. scMP-12 or scMP-12-mutNSs that underwent two rounds of amplification (P2 samples) in Vero-G cells, in which culture fluids from the infected Vero-G cells were collected at 5 to 7 days post-inoculation, was used for the studies. All the mutations introduced into scMP-12 and scMP-12-mutNSs were confirmed by sequencing of viral genomes obtained from the P2 samples.

### Plaque assay

Infectivity of MP-12 and ZH501 was determined in Vero-G cells and VeroE6 cells, respectively, by a standard plaque assay^48^. For determining the infectivity of scMP-12 and scMP-12-mutNSs, infected Vero-G cells were stained by anti-N antibody as described previously^45^.

### Antibodies and Western blot analysis

Dilution of the antibodies are shown in parentheses. Goat anti-β-actin polyclonal (1:5000) and horseradish peroxidase (HRP) conjugated goat anti-mouse IgG (1:10,000) antibodies were purchased from Santa Cruz Biotechnology. HRP conjugated horse anti-mouse IgG (1:10,000), HRP conjugated goat anti-rabbit IgG (1:10,000), and rabbit anti-PKR polyclonal antibody (1:1,000) were purchased from Cell Signaling Technology. Anti-MP-12 (1:3,000) was a gift from Dr. Robert Tesh at the University of Texas Medical Branch. Samples were prepared by suspending the cells with 2x sample buffer and then boiling for 5 min. An equal volume of samples were applied onto SDS-polyacrylamide gel for electrophoresis and separated proteins were transferred onto polyvinylidene difluoride membranes (Bio-Rad). The membranes were blocked with 3% nonfat milk for 1 h and incubated with the primary antibody for 1 h at room temperature or for overnight at 4 °C, following incubation with the secondary antibody for 1 h at room temperature. The ECL Western Blotting Detection Kit (GE Healthcare) was used for detection of Western blots. All of the original uncropped images of Western blots are shown in Supplementary Figures 3 and 5.

### Northern blot analysis

TRIzol reagent (Invitrogen) was used for extraction of RNAs prior to Northern blot analysis, as described previously^50^. A digoxigenin RNA labeling kit (Roche) was used to generate viral-sense-specific RNA probes, as described previously^48^. Digoxigenin wash and block buffer set (Roche) was used for the detection of viral RNAs by following the manufacturer’s protocol. All of the original uncropped images of Northern blots are shown in Supplementary Figures 1, 2 and 4. The RNA probes for detection of genomic sense viral RNA hybridized with L RNA at nucleotide positions 19–756, M RNA at nucleotide positions 1297–2102, and S RNA at nucleotide positions 39–776 from the 3′ ends, respectively. The probe for detection of anti-genomic sense S RNA binds to S RNA and N mRNA at nucleotide positions 39–776 from the 5′ end.

### 5EU labeling of newly synthesized RNA

RNA synthesis in infected cells was analyzed by using Click-iT RNA Alexa Fluor 488 Imaging Kit (Thermo Fisher Scientific) as described in^42^. Briefly, VeroE6 cells were infected with scMP-12-mutNSs at a multiplicity of infection (moi) of 1 and treated with 5EU for 1 h at 16 h post-infection. As controls, cells infected with MP-12 or MP-12ΔNSs and those treated with actinomycin D were used. To prepare actinomycin D-treated cells, cells were incubated with 5 μg/ml of actinomycin D for 30 min prior to 5EU treatment and during the 5EU treatment. After 1 h incubation with 5EU, cells were fixed with 4% paraformaldehyde and treated with fluorescent azide to visualize labeled RNA by click chemistry. Cells were further incubated with anti-N rabbit polyclonal antibody (1:500)^45^ followed by Alexa Fluor 594 conjugated anti-rabbit antibody (1:500) to stain RVFV N protein. Samples were analyzed with an Olympus BX65 fluorescent microscope.

### Immunization and RVFV challenge

Five-week-old female CD1 mice were intramuscularly immunized with 10^4^ PFU of MP-12, 10^5^ or 10^4^ PFU of scMP-12, or 10^5^ or 10^4^ PFU of scMP-12-mutNSs (n = 15 for 10^4^ PFU of scMP-12 and scMP-12-mutNSs, n = 10 for other groups). A total of 100 μl of sample at concentration of 10^5^ or 10^6^ PFU/ml was injected into each mouse. After immunization, the remaining inoculum was subjected to plaque assay to confirm the immunization dose. Blood was collected from the retro-orbital venous plexus of the mice at 36 day post-immunization for serum isolation. Forty days post-vaccination, the immunized mice were challenged intraperitoneally with the virulent RVFV strain ZH501 at dose of 10^3^ PFU, which was equivalent to approximately 200 times the 50% minimal lethal dose (LD_50_)^58^ and monitored for survival and clinical signs of disease for 21 days post-challenge. The grade of clinical disease was scored as follows: 1- healthy; 2- lethargic; 3- ruffled fur, lethargic, hunched posture, orbital tightening; 4- reluctance to move when stimulated, paralysis, unable to access feed and water normally, moribund appearance or ≥20% weight loss. Animals that were assigned a score of 4 were immediately euthanized for humane reasons and were reported as dead the following day.

### Virus neutralization assay

Neutralizing antibody titers in serum were measured by using PRNT80, as previously described^59^.

### N IgG ELISA

ELISA was performed as described in^52^ with minor modifications. Purified RVFV N protein (MyBioSource) was added to 96-well ELISA plates at a concentration of 100 ng/well. Following overnight incubation at 4 °C, the plates were washed 3 times with PBS containing 0.05% Tween 20 (PBS-T) and incubated with blocking buffer (PBS-T containing 0.5% bovine serum albumin) at 37 °C for 2 h. After removing the blocking buffer, serum samples were added into the wells and incubated at 37 °C for 1 h. The wells were washed 4 times with PBS-T and incubated with horseradish peroxidase-conjugated horse anti-mouse IgG (Cell signaling) at 37 °C for 1 h. After washing with PBS-T four times, 2,2′-azino-bis [3-ethylbenziazoline-6-sulfonic acid] (ABTS) was added to the wells. The plate was incubated at room temperature for 30 min, and the optical density at 405 nm was recorded. The cutoff value was defined as the mean plus 2 times the standard deviation of 10 HBSS inoculated mouse serum samples (1:400) for anti-N IgG. The highest dilution of sera that gave an OD value larger than the cutoff was designated the anti-N antibody titer.

## Electronic supplementary material

Supplementary information

## Data Availability

The datasets generated during and/or analyzed during the current study are available from the corresponding author upon reasonable request.
