# Supplementary material for: A single-cycle replicable Rift Valley fever phlebovirus vaccine carrying a mutated NSs confers full protection from lethal challenge in mice
Source: Sci Rep. 2018 Nov 20;8:17097. doi: 10.1038/s41598-018-35472-7 (PMC6244155; doi:10.1038/s41598-018-35472-7)
Supplement: Supplementary file 1 — Supplementary information [file 41598_2018_35472_MOESM1_ESM.pdf]

Supplementary information for

A single-cycle replicable Rift Valley fever phlebovirus vaccine carrying a mutated NSs confers full protection from lethal challenge in mice.

Kaori Terasaki, Terry L Juelich, Jennifer K Smith, Birte Kalveram, David D Perez, Alexander N Freiberg, and Shinji Makino

*Corresponding author: Shinji Makino
shmakino@utmb.edu

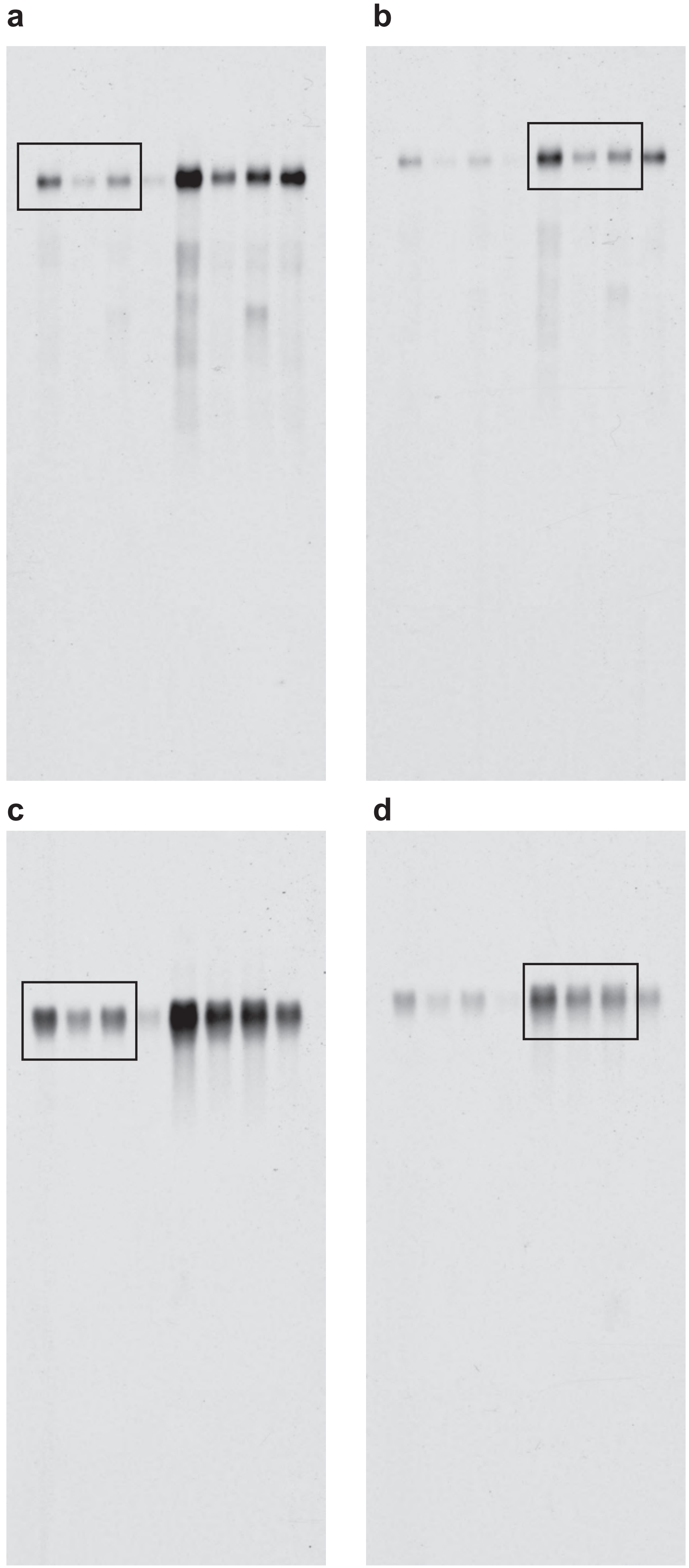

Supplementary Figure 1. Uncropped images of Northen blots. (a) For Fig. 1d. L RNA in Vero-G cells (top left panel). (b) For Fig. 1d. L RNA in BHK cells (top right panel). (c) For Fig. 1d. M RNA in Vero-G cells (second left panel from the top). (d) For Fig. 1d. M RNA in BHK cells (second right panel from the top).

a

b

c

d

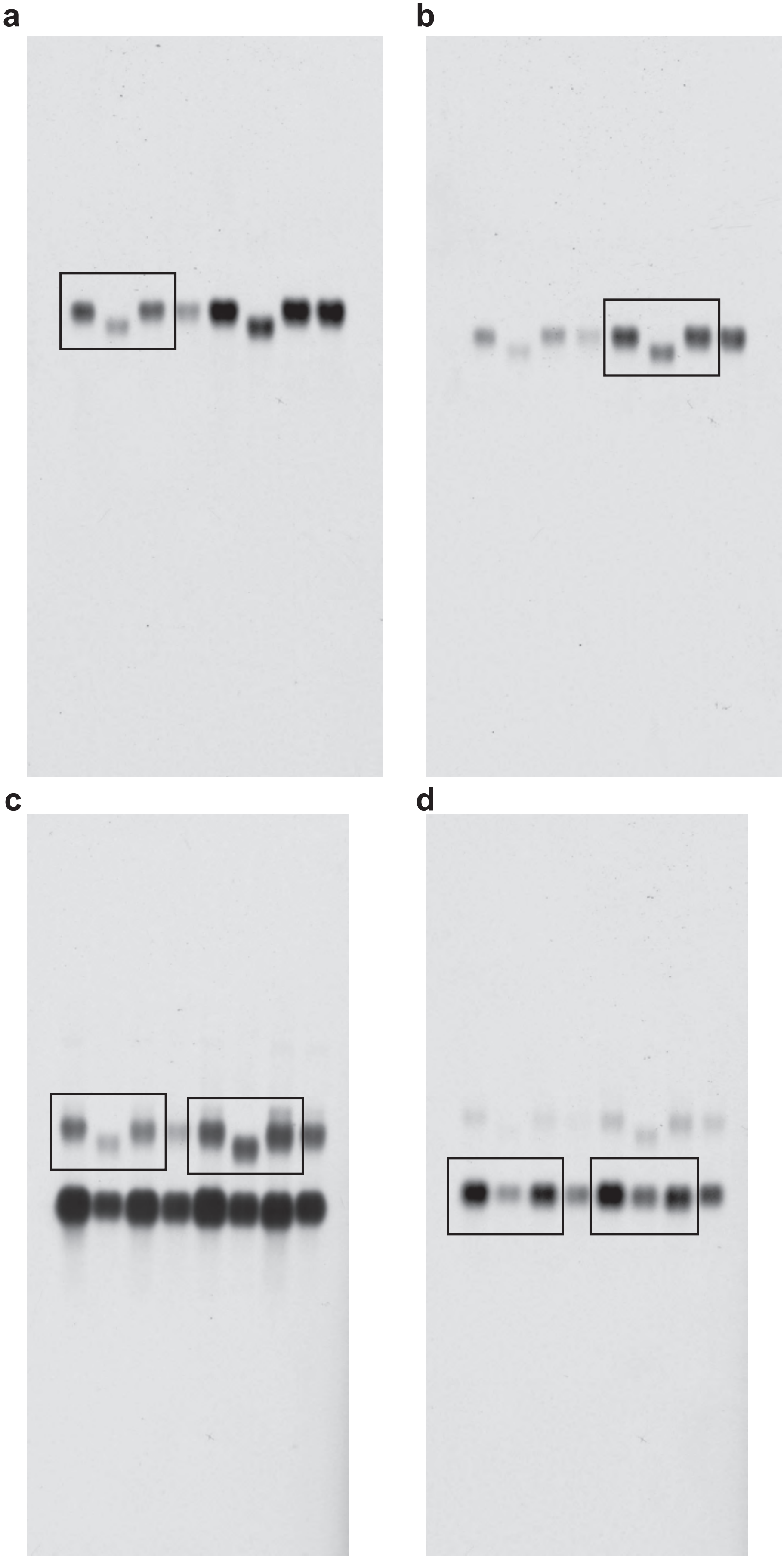

Supplementary Figure 2. Uncropped images of Northen blots. (a) For Fig. 1d. S RNA in Vero-G cells (third left panel from the top). (b) For Fig. 1d. S RNA in BHK cells (third right panel from the top). (c) For Fig. 1d. anti-genomic sense S RNA (forth panels from the top). (d) For Fig. 1d. N mRNA (bottom panels).

a

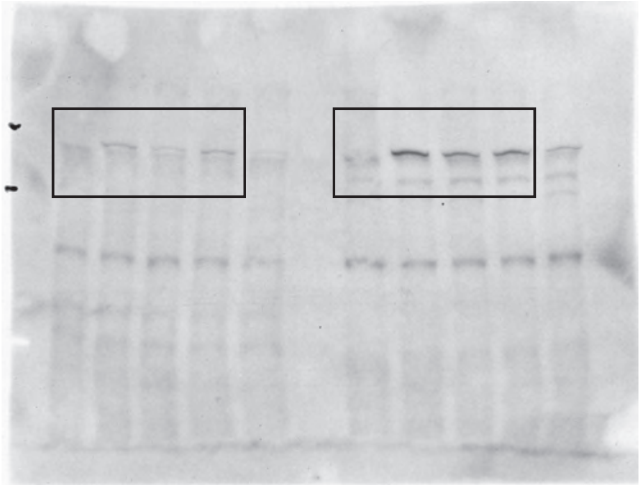

b

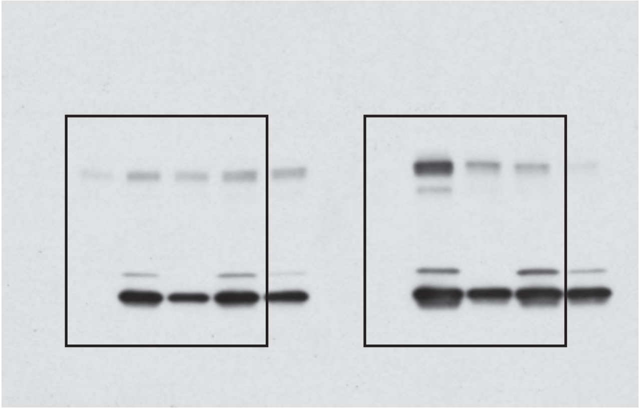

c

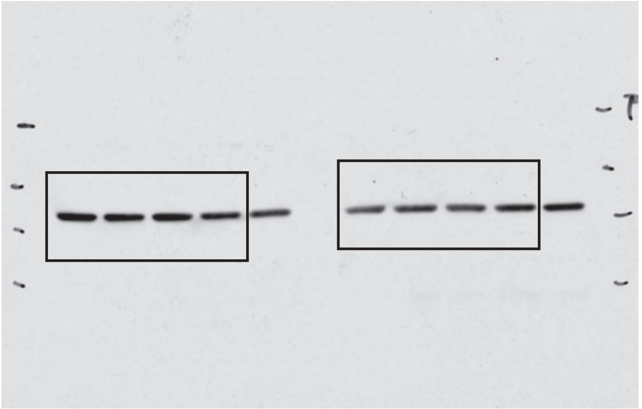

Supplementary Figure 3. Uncropped images of Western blots. (a) For Fig. 1e. L protein (top panels). (b) For Fig. 1e. Gn/Gc, NSs and N protein (middle panels). (c) For Fig. 1e. beta-actin (bottom panels).

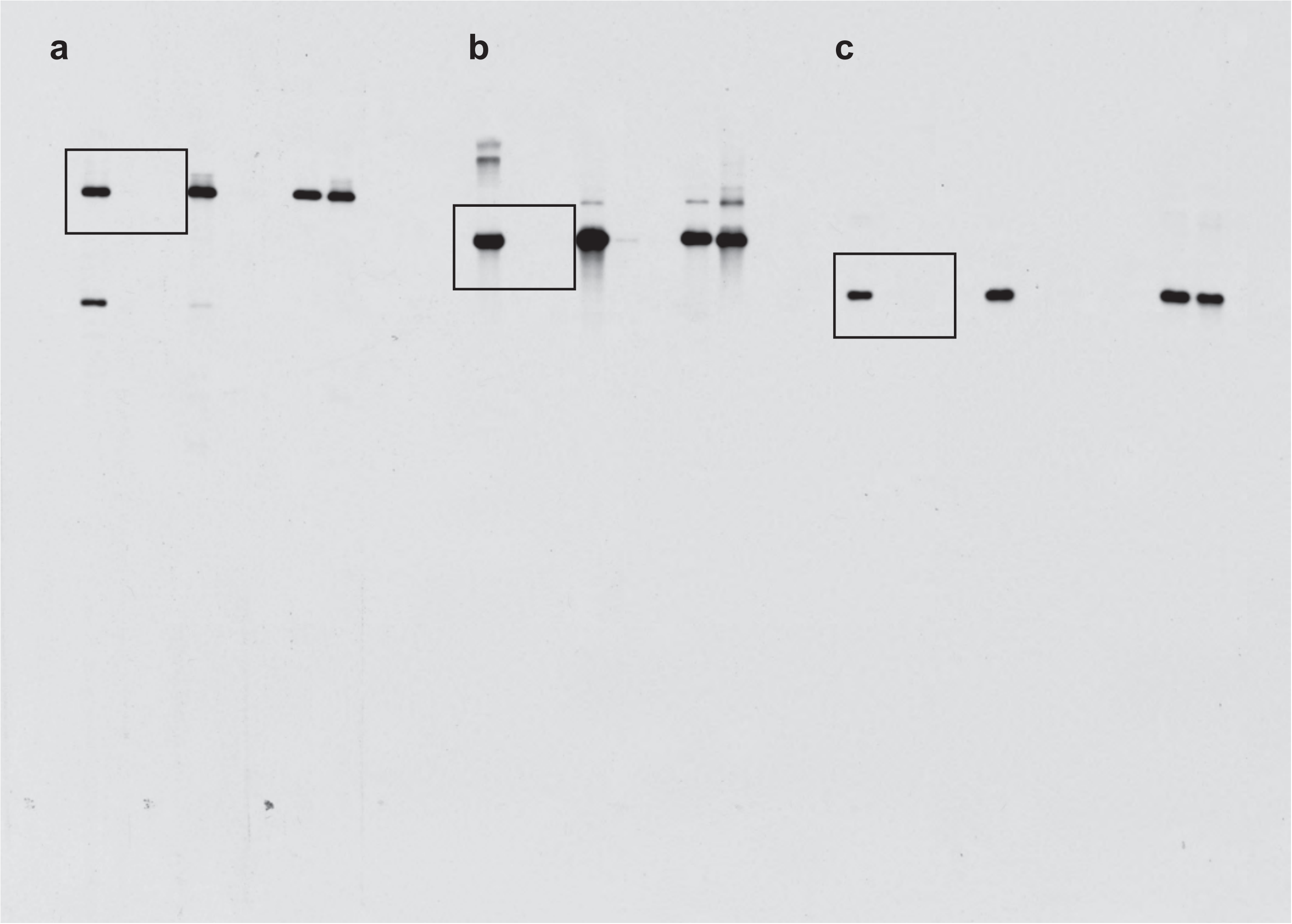

Supplementary Figure 4. Uncropped images of Northern blots. (a) For Fig. 1f. L RNA (top panel). (b) For Fig. 1f. M RNA (middle panel). (c) For Fig. 1f. S RNA (bottom panel).

a

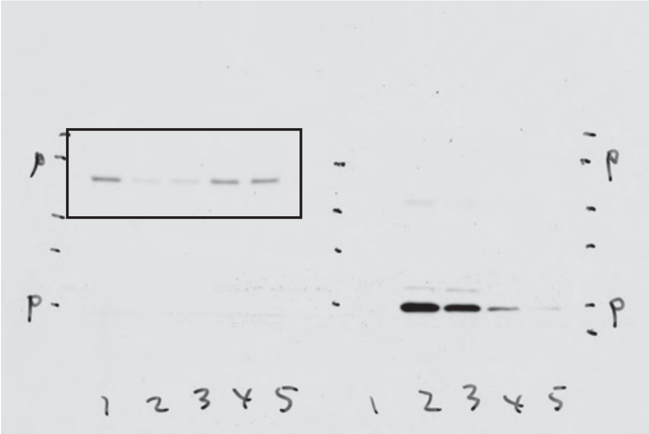

b

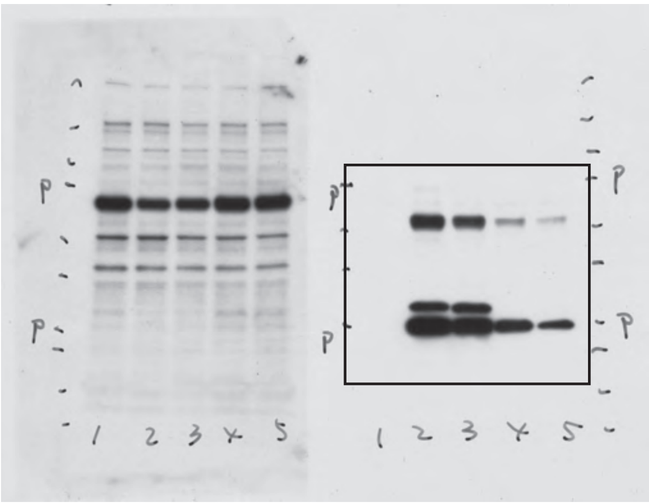

c

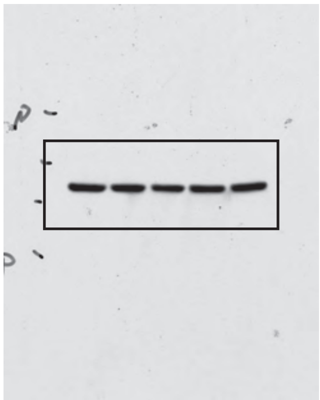

Supplementary Figure 5. Uncropped images of Western blots. (a) For Fig. 2. PKR (top panel). (b) For Fig. 2. Gn/Gc, NSs and N protein (middle panel). (c) For Fig. 2. beta-actin (bottom panel).
